# Changes in Corneal Deformation Parameters after Lenticule Creation and Extraction during Small Incision Lenticule Extraction (SMILE) Procedure

**DOI:** 10.1371/journal.pone.0103893

**Published:** 2014-08-14

**Authors:** Yang Shen, Jing Zhao, Peijun Yao, Huamao Miao, Lingling Niu, Xiaoying Wang, Xingtao Zhou

**Affiliations:** Key Lab of Myopia, Ministry of Health, Department of Ophthalmology, EYE & ENT Hospital of Fudan University, Shanghai, China; Casey Eye Institute, United States of America

## Abstract

**Purpose:**

To investigate the effects of lenticule creation and subsequent corneal lenticule extraction on corneal deformation parameters during small incision lenticule extraction (SMILE) procedure.

**Materials and Methods:**

In this prospective study, 18 eyes of 10 patients (27.90±7.11 years, −5.64±2.45 diopters) scheduled for SMILE procedure were enrolled. Changes in the corneal deformation parameters, including deformation amplitude (DA), applanation time(AT1 and AT2), applanation length(AL1 and AL2), corneal velocity(CV1 and CV2), peak distance(P.Dist.), radius and intraocular pressure values were measured preoperatively, immediately after lenticule creation and subsequent to corneal lenticule extraction in all eyes with the Corvis Scheimpflug Technology (Corvis ST, OCULUS, Wetzlar, Germany). Repeated measures analysis of variance (ANOVA) with bonferroni-adjusted post hoc comparisons was performed to investigate changes following each step of the procedure.

**Results:**

All surgical procedures were uneventful. A significant difference was detected among the three time points (pre-operation, post-lenticule creation and post lenticule extraction) for AT1 (P<0.001), AT2 (P = 0.001), DA(P<0.001), and IOP(P = 0.002). Bonferroni-adjusted post hoc comparisons indicated that there was no significant change in AT1, AT2, DA, or IOP after lenticule creation (post hoc P>0.05), but there was a significant change in these parameters following subsequent corneal lenticule extraction (post hoc P<0.01), when compared to values obtained pre-operatively. The scheimpflug camera of the Corvis ST demonstrated the intralamellar small gas bubbles formed from the vaporisation of tissue after lenticule creation and a gray zone was observed between the cap and the residual stromal bed after lenticule extraction.

**Conclusions:**

There is a significant change in corneal deformation parameters following SMILE procedure. The changes may be caused predominantly by stromal lenticule extraction, while lenticule creation with femtosecond laser may not have an obvious effect on corneal deformation properties.

## Introduction

Currently, femtosecond laser has found an increasingly wide utilization in refractive surgeries including femtosecond laser-assisted laser in situ keratomileusis (FS-LASIK) [1], femtosecond lenticule extraction (FLEx) [2] and small incision lenticule extraction (SMILE) [3] procedures. However, the safety of corneal refractive surgeries has attracted more and more attention, not only because of the importance in predicting outcomes but also the necessity of assessing operation risks. Uzbek [4] investigated the changes in biomechanical properties after flap creation using femtosecond laser and subsequent excimer laser ablation with an ocular response analyzer (ORA). He found a significant decrease in Peak 1 after both flap creation and after ablation. This indicated that a change of biomechanical property due to flap creation does occur, specifically a reduction in stiffness. Significant reductions of corneal hysteresis (CH) and corneal resistance factor (CRF) were only detected after excimer laser ablation. He proposed that the major biomechanical consequence of intra-LASIK is in the ablation, while flap creation also made a contribution to corneal biomechanical compromise.

**Figure 1:**
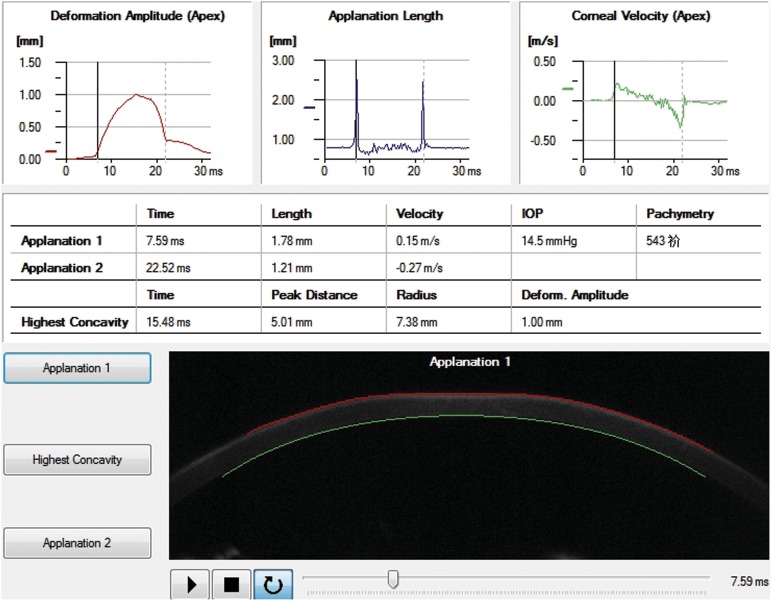
The real-time corneal deformation parameters of a participant recorded when the cornea was just flattened.

**Figure 2:**
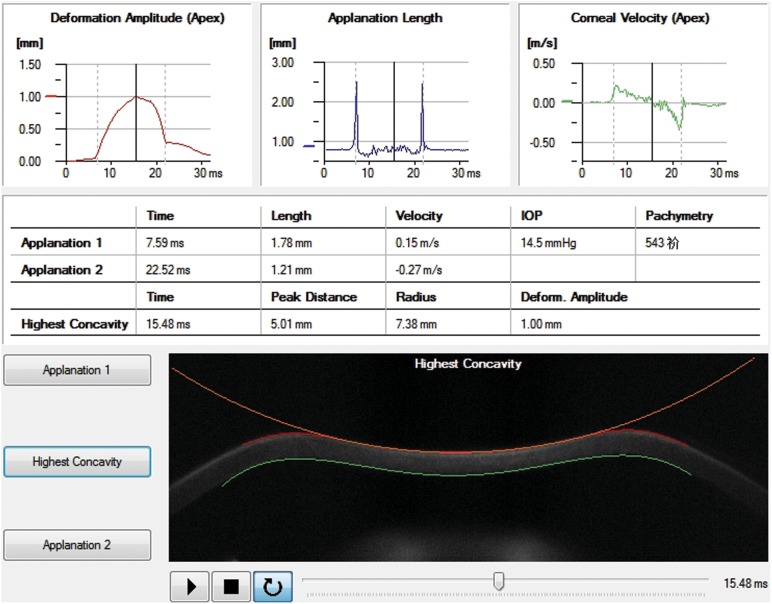
The real-time corneal deformation parameters of a participant recorded when the cornea was flattened to the highest concavity.

**Figure 3:**
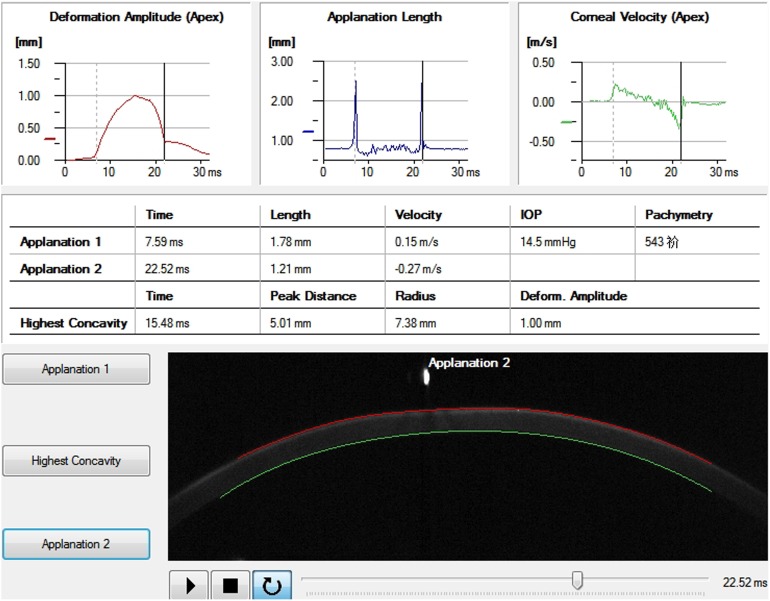
The real-time deformation parameters of a participant recorded when the cornea recovered to its original shape.

Recently, a novel non-contact tonometer Corvis Scheimpflug Technology (Corvis ST, OCULUS, Wetzlar, Germany), which uses a high-speed Scheimpflug camera to capture the dynamic process of corneal deformation caused by an air puff, has become a useful instrument for evaluating the biomechanical properties, (Figure 1–3). By calculating the deformation parameters (including the length, velocity and amplitude of the corneal concavity) resulting from the air puff, changes of corneal biomechanical properties can be evaluated. Corvis ST produces more parameters than ORA does for biomechanical analysis. It has been used to investigate corneal biomechanical changes in glaucoma patients. [5] However, the study on changes of corneal deformation parameters measured with Corvis ST during refractive surgeries has not been reported yet.

Small incision lenticule extraction (SMILE) is a new, flapless, minimal invasive and all-in-one refractive procedure, which can correct myopia without creating a corneal flap. A femtosecond laser is used to create a refractive stromal lenticule, which can be removed from a small peripheral incision, and therefore Bowman’s layer is intact for the most part postoperatively. However, up to present, our knowledge about the changes in biomechanical properties following SMILE procedure is still limited. In this prospective study, we improved Uzbek’ work. We measured the corneal deformation parameters with Corvis ST preoperatively, immediately after lenticule creation and subsequently to lenticule extraction during SMILE procedure to investigate the change of corneal biomechanical properties as a response to lenticule creation versus subsequent lenticule extraction [6]–[7].

## Materials and Methods

### Ethics Statement

This study was approved by ethics committee of the Eye and ENT Hospital, Fudan University, and was carried out with due regard to the tenets of the Declaration of Helsinki. After a detailed explanation of the procedures, written informed consent was obtained from all participants.

### Participants

This was a prospective self-controlled study.18 eyes of 10 myopic patients (5 males and 5 females) without a previous history of an ocular disorder were enrolled. The mean age was 27.90±7.11 years and pre-operative spherical equivalent (SE) was −5.64±2.45 diopters (D) (Table 1).

**Table 1:** Demographic Data.

Total Subjects n = 18	Mean	Standard Deviation	Range
Age, year	27.90	7.11	20∼39
Pre-operative MRSE, D	−5.64	2.45	−9.75∼−2.38
Post-operative MRSE, D	0.10	0.39	−0.88∼0.50
Pre-operative CCT, µm(CST)	525.83	20.93	482∼551
Post-operative CCT, µm(CST)	442.89	47.32	341∼518
Pre-operative CCT, µm(PHR)	546.83	18.59	510∼576
Post-operative CCT, µm(PHR)	438.61	37.53	392∼506

MRSE = Manifest refraction spherical equivalent; CCT = Central corneal thickness; CST = Corvis ST; PHR = Pentacam HR.

### Ophthalmologic Examinations

Each patient underwent routine pre-operative ophthalmologic examinations, including uncorrected distance visual acuity (UDVA), manifest refraction, corrected distance visual acuity (CDVA), slit lamp examination and fundus examination.

Corneal deformation parameters were acquired with the Corvis ST preoperatively, immediately after the lenticule creation (after laser scanning but prior to lenticule extraction) and immediately after corneal lenticule extraction. The patient was asked to fixate ahead on a target (a red light) in the tonometer with his or her chin on a chin-rest and forehead against the forehead strap. A monitor on the tonometer would guide the operator aim the nozzle at the center of the patient’s cornea with a joystick attached to the tonometer and it puffed an air jet onto the center of the cornea automatically. Then the process of corneal deformation caused by the air puff was recorded. Meanwhile, the Corvis ST automatically calculated the applanation time, applanation length and corneal velocity while the cornea was flattened for the first time (applanation 1) (Figure 1), depressed to the highest concavity (Figure 2) and applanated for the second time during the recovery from the air puff (applanation 2) (Figure 3). The corneal deformation amplitude (DA), peak distance (P.Dist.), radius, central corneal thickness (CCT) and intraocular pressure (IOP) values were also calculated and recorded.

### Surgical Techniques

SMILE procedures were performed with the VisuMax femtosecond laser system (Carl Zeiss Meditec AG, Germany), under topical anesthesia using three drops of 0.4% Oxybuprocaine Hydrochloride (Santen Pharmaceutical Co., Japan). Femtosecond laser repetition rate was 500 kHz and pulse energy of 130 nJ was used to perform surgical refractive corrections for the patients. The intended thickness of the cap was set to 110 µm, and its diameter was set to 7.5 mm. The width of the side cut was set to 2 mm in the 12 o’clock position. After the lenticule was successfully created, the patient was asked to sit up to have the second Corvis ST measurement immediately. After the manual removal of the lenticule, the final Corvis ST measurement was acquired immediately.

### Data Analysis and Statistical Evaluation

Statistical analysis was performed using SPSS 19 software (SPSS Inc., IBM, USA). Repeated measures analysis of variance (ANOVA) with bonferroni-adjusted post hoc comparisons was performed to evaluate differences between pre-operation and post-lenticule creation as well as post-lenticule extraction. Cut-off P values were 0.05.

## Results

All surgical procedures were uneventful. No complications were observed.

The main corneal deformation parameters measured pre-operative, immediately after lenticule creation and after lenticule extraction were listed in Table 2.

**Table 2:** The main corneal deformation parameters.

Total Subjects n = 18	Pre-operation (1)	Post-lenticule creation (2)	Post-lenticule extraction (3)
Applanation time (applanation 1), ms	7.65±0.19	7.48±0.34	7.26±0.21
F = 14.373, P**<0.001			
(1) vs (2) post hoc P = 0.202; (1) vs (3) post hoc P**<0.001; (2) vs (3) post hoc P* = 0.028			
Applanation time (highest concavity), ms	16.88±0.71	17.04±0.63	17.31±0.54
F = 2.966, P = 0.065			
Applanation time (applanation 2), ms	22.45±0.33	22.38±0.73	22.85±0.40
F = 7.973, P** = 0.001			
(1) vs (2) post hoc P = 1.000; (1) vs (3) post hoc P** = 0.002; (2) vs (3) post hoc P* = 0.012			
Applanation length (applanation 1), mm	1.75±0.30	1.92±0.31	1.88±0.30
F = 1.791, P = 0.182			
Applanation length (applanation 2), mm	1.65±0.39	1.75±0.55	1.40±0.52
F = 2.427, P = 0.103			
Peak distance, mm	4.98±0.26	5.00±0.49	5.18±0.71
F = 1.260, P = 0.296			
Corneal velocity (applanation 1), m/s	0.14±0.03	0.15±0.05	0.15±0.04
F = 0.163, P = 0.851			
Corneal velocity (applanation 2), m/s	−0.38±0.08	−0.37±0.16	−0.49±0.10
F = 4.820, P* = 0.014			
(1) vs (2), post hoc P = 1.000; (1) vs (3), post hoc P** = 0.006; (2) vs (3), post hoc P = 0.061			
Radius, mm	7.12±0.58	7.04±2.01	5.88±0.48
F = 4.920, P* = 0.037			
(1) vs (2), post hoc P = 1.000; (1) vs (3), post hoc P**<0.001; (2) vs (3), post hoc P = 0.093			
Deformation amplitude, mm	1.04±0.07	1.07±0.10	1.18±0.10
F = 20.402, P**<0.001			
(1) vs (2), post hoc P = 0.520; (1) vs (3), post hoc P**<0.001; (2) vs (3), post hoc P** = 0.001			
Intraocular pressure, mmHg	14.94±1.28	15.69±3.34	12.72±1.24
F = 10.778, P** = 0.002			
(1) vs (2), post hoc P = 0.998; (1) vs (3), post hoc P**<0.001; (2) vs (3), post hoc P** = 0.005			

Repeated measures analysis of variance (ANOVA) with bonferroni-adjusted post hoc comparisons.

P*<0.05, P**<0.01.

**Figure 4:**
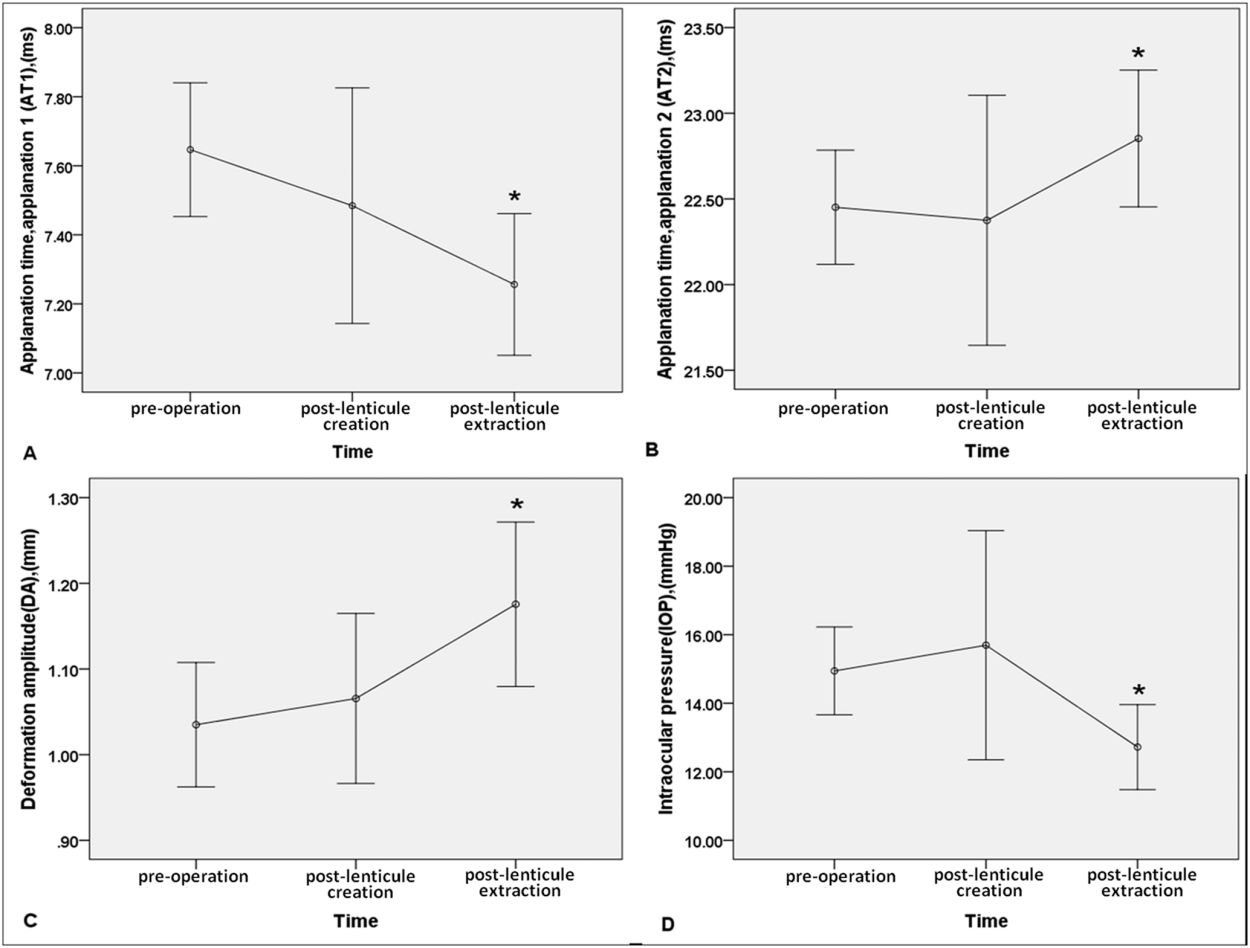
A. The difference in mean value of AT1 measured preoperative, after lenticule creation, and after lenticule extraction during SMILE. The mean value of AT1 after lenticule extraction was significantly lower than that before surgery and that after lenticule creation (post hoc P<0.001 and post hoc P = 0.028, respectively), while the decrease in AT1 after lenticule creation was not significant (post hoc P = 0.202) when compared to the values before surgery. **B. The difference in mean value of AT2 measured preoperative, after lenticule creation, and after lenticule extraction during SMILE.** The mean value of AT2 after lenticule extraction was significantly higher than that before surgery and that after lenticule creation (post hoc P = 0.002 and post hoc P = 0.012, respectively), while the increase in AT2 after lenticule creation was not significant (post hoc P = 1.000) when compared to the values before surgery. **C. The difference in mean value of DA measured preoperative, after lenticule creation, and after lenticule extraction during SMILE.** The mean value of DA after lenticule extraction was significantly higher than that before surgery and that after lenticule creation (post hoc P<0.001 and post hoc P = 0.001, respectively), while the increase in DA after lenticule creation was not significant (post hoc P = 0.520) when compared to the values before surgery. **D. The difference in mean value of IOP measured preoperative, after lenticule creation, and after lenticule extraction during SMILE.** The mean value of IOP after lenticule extraction was significantly lower than that before surgery and that after lenticule creation (post hoc P<0.001 and post hoc P = 0.005, respectively), while the increase in DA after lenticule creation was not significant (post hoc P = 0.998) when compared to the values before surgery.

Mean AT1 was 7.65±0.19 milliseconds (ms), 7.48±0.34 ms, and 7.26±0.21 ms preoperative, after lenticule creation and after lenticule extraction, respectively. The repeated measures ANOVA (n = 18) indicated a significant difference among the three time points (F = 14.373, P<0.001). The post hoc comparison test demonstrated that the decrease in AT1 after lenticule creation was not significant (post hoc P = 0.202) when compared to the values before surgery. However, the decrease in AT1 after lenticule extraction was significant when compared to the values before surgery (post hoc P<0.001) and after lenticule creation (post hoc P = 0.028) (Figure 4A).

Mean AT2 was 22.45±0.33 ms, 22.38±0.73 ms, and 22.85±0.40 ms at the three time points, respectively. The repeated measures ANOVA indicated a significant difference among these time points (F = 7.973, P = 0.001). The post hoc comparison test demonstrated that the change in mean AT2 value after lenticule creation was not significant (post hoc P = 1.000), but the increase in AT2 after lenticule extraction was significant when compared to the values before surgery (post hoc P = 0.002) and after lenticule creation (post hoc P = 0.012) (Figure 4B). The Pearson’s Correlation analysis revealed a significant negative correlation between AT1 and AT2 before (r = −0.866, P<0.001) and after (r = −0.795, P<0.001) surgeries.

Mean DA was 1.04±0.07 mm, 1.07±0.10 mm, and 1.18±0.10 mm at the three time points, respectively. The repeated measures ANOVA indicated a significant difference among the three time points (F = 20.402, P<0.001). The post hoc comparison tests demonstrated that the increase in DA after lenticule creation was not significant (post hoc P = 0.520), but the increase in DA after lenticule extraction was significant when compared to the values before surgery (post hoc P<0.001) and after lenticule creation (post hoc P = 0.001) (Figure 4C).

Mean IOP were 14.94±1.28 mmHg, 15.69±3.34 mmHg, and 12.72±1.24 mmHg, respectively. The repeated measures ANOVA indicated a significant difference among the three time points (F = 10.778, P = 0.002). The post hoc comparison test demonstrated that the decrease in mean IOP value after lenticule creation was not significant (post hoc P = 0.998), but the reduction in IOP after lenticule extraction was significant when compared to the values before surgery (post hoc P<0.001) and after lenticule creation (post hoc P = 0.005) (Figure 4D).

**Figure 5:**
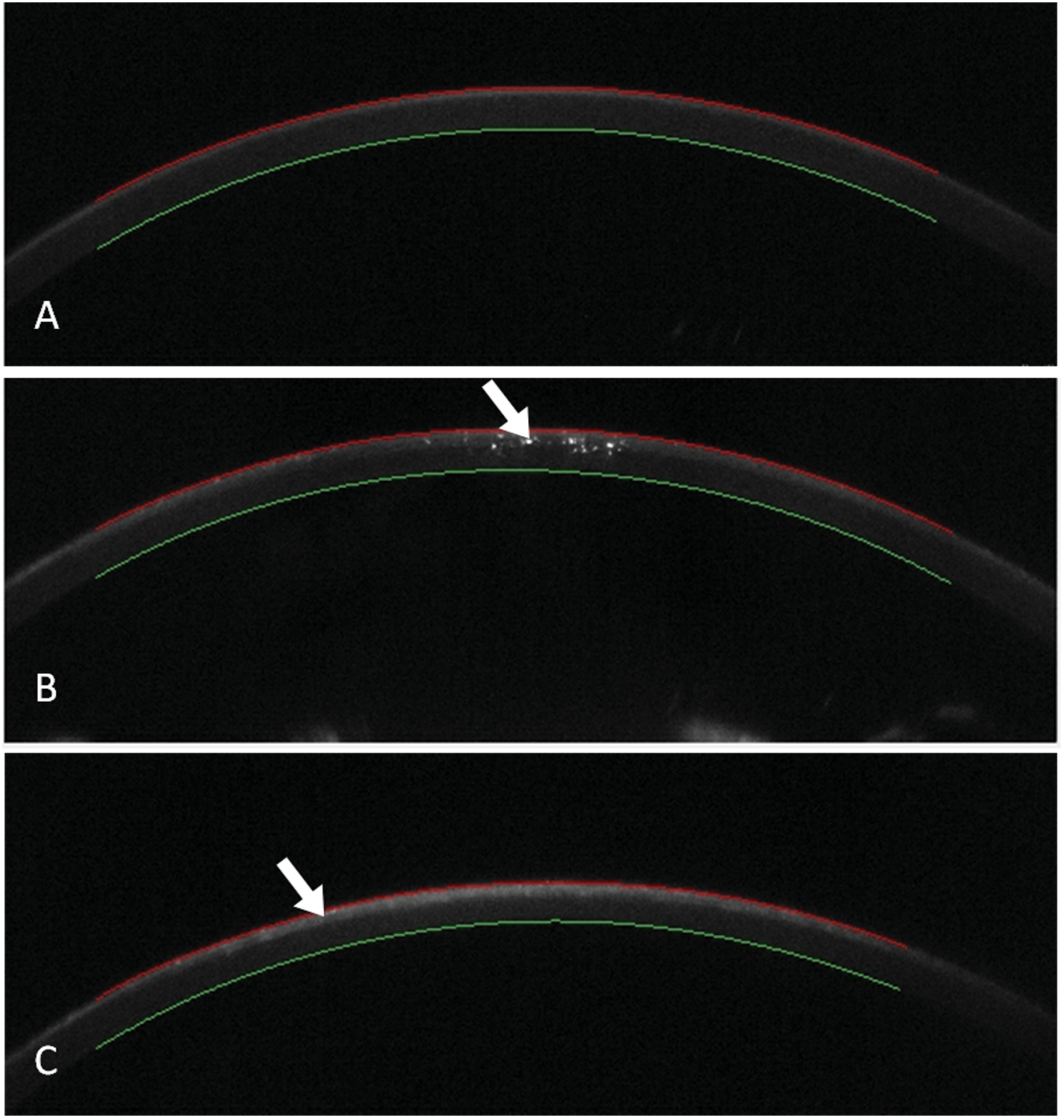
A. The anterior segment of the eye (before surgery) captured by the Scheimpflug camera. The cornea was clear. **B. The anterior segment of the eye (after lenticule creation prior to lenticule extraction).** The bright spot marked by the while arrow was the intralamellar small gas bubble formed from the vaporisation of tissue during lenticule creation. **C. The anterior segment of the eye (after lenticule extraction).** The gray area marked by the while arrow may be the potential gap between the cap and the residual stromal bed from where the refractive lenticule was extracted.

The intralamellar small gas bubbles formed from the vaporisation of tissue after lenticule creation and a gap-like gray zone between the cap and the residual stromal bed after lenticule extraction were observed by the scheimpflug camera of the Corvis ST (Figure 5A–C).

## Discussion

The Corvis ST allows a measurement in vivo of the corneal deformation properties referred to as DA, AT1 and AT2 in addition to IOP, which appear to be indicators of the overall biomechanical properties of the cornea. Hon Y reported that DA, AT1, AT2 and IOP values were well repeatable while corneal velocities, applanation lengths and time at the highest concavity were not. [7]–[8] So far, Corvis ST has not clinically used in the field of refractive surgeries. As the latest and minimally invasive refractive procedure, SMILE should be outstanding in maintaining corneal biomechanical stability. However the studies on the changes in biomechanical properties following SMILE procedure are still limited. Thus, we conduct this prospective study to investigate the changes of corneal deformation properties during SMILE procedure as a response to lenticule creation versus in response to subsequent lenticule extraction.

Our study showed that the increase in DA after lenticule creation was not significant, whereas that after lenticule extraction was significant when compared to the values before surgery and after lenticule creation. DA refers to the maximum amplitude when the cornea is flatten to its highest curvature by the air jet puffed from the Corvis ST and is regarded as a parameter which reflects corneal stiffness. [8]–[9] Hon Y [8] reported that a thinner cornea was associated with a higher DA. In this regard, our result may indicate that the corneal stiffness did not change significantly after lenticule creation with the VisuMax femtosecond laser system. The major reason for corneal stiffness reduction may be the reduction in corneal thickness as the DA value increased significantly immediately after the lenticule was extracted. Besides, the Scheimpflug camera of the Corvis ST demonstrated the intralamellar small gas bubbles formed from the vaporisation of tissue after lenticule creation and a gap-like gray zone between the cap and the residual stromal bed after lenticule extraction (Figure 5C). The gap-like gray zone may partially contribute to the overall compromise of the corneal stiffness.

In this study we found that AT1 and AT2 did not change significantly after lenticule creation, while changes of AT1 and AT2 were significant after lenticule extraction. AT1 refers to the time from start until the cornea was flattened for the first time (applanation 1) (Figure 1), while AT2 refers to the time from start until the cornea rebounds to flattened status (Figure 3) from its highest concavity (Figure 2). In addition, significant negative correlations between AT1 and AT2 before and after surgeries were both detected. This indicated that the sooner the cornea was flattened, the later the original shape would recover. Luce [10] and Ambrósio R Jr [11] reported similar results in normal population by using ORA. And our previous study also detected similar results in those who underwent refractive surgery by using Corvis ST. [12] However, the changes in corneal applanation time after lenticule extraction during SMILE procedure has not been reported yet.

IOP value always decreases after corneal refractive surgeries because of flap creation or corneal tissue ablation. Several previous studies have confirmed that IOP values decreased more significantly after thicker flap creation or deeper stromal ablation. [13] Pepose JS [14] reported that IOP was filtered through the biomechanical signature of the cornea and variable affected by corneal surgery. In the current study, IOP values did not change significantly after lenticule creation but decreased significantly after lenticule extraction. This further indicated that lenticule extraction predominantly compromised corneal biomechanical properties as partial corneal tissue was removed, while lenticule creation did not significantly influence the biomechanical properties. Several in-vivo and in-vitro studies showed that IOP value would dramatically increase during femtosecond laser assisted corneal refractive surgeries as the response to attachment and suction. [15]–[16] In this study we did not detect a significant increase in IOP after lenticule creation, implying that the IOP rise might be temporary. And the SMILE procedure is safe.

It is interesting that DA, AT1, AT2 and IOP did not change significantly after lenticule creation during SMILE procedure, but the value of Peak 1 do change after cutting a flap during FS-LASIK in earlier studies [4]. This indicated that SMILE may outplay FS-LASIK at operative safety and maintaining corneal biomechanical stability. A possible explanation is that flap is created by cutting through the corneal epithelium and Bowman’s layer. This process breaks all the corneal collagen fibers between the flap and the stromal bed. However, SMILE procedure does not create a complete LASIK flap, and the process of lenticule creation does not cut Bowman’s layer, and thus maintains the structural integrity of the cornea. We hypothesize that if SMILE procedure was suspend for some reason during the process of lenticule creation (before lenticule extraction), the corneal biomechanical properties may not change significantly.

The present study had some limitations. First, the relatively small sample size made it difficult to completely avoid a possible selection bias. Second, a small quantity of gas bubbles is still present after lenticule creation, which may leads to some confounding circumstances. However, both the mean values and the standard deviation values of these parameters did not change dramatically after lenticule creation (Table 2). So these gas bubbles should have a limited influence only.

In conclusion, there is a significant change in corneal deformation parameters following SMILE procedure. The changes may be caused predominantly by stromal lenticule extraction, while lenticule creation with femtosecond laser may not have an obvious effect on corneal deformation properties.
